# Engineering PQS Biosynthesis Pathway for Enhancement of Bioelectricity Production in *Pseudomonas aeruginosa* Microbial Fuel Cells

**DOI:** 10.1371/journal.pone.0063129

**Published:** 2013-05-20

**Authors:** Victor Bochuan Wang, Song-Lin Chua, Bin Cao, Thomas Seviour, Victor J. Nesatyy, Enrico Marsili, Staffan Kjelleberg, Michael Givskov, Tim Tolker-Nielsen, Hao Song, Joachim Say Chye Loo, Liang Yang

**Affiliations:** 1 Singapore Centre on Environmental Life Sciences Engineering (SCELSE), Nanyang Technological University, Singapore, Singapore; 2 Singapore Centre on Environmental Life Sciences Engineering (SCELSE), National University of Singapore, Singapore, Singapore; 3 School of Biological Sciences, Nanyang Technological University, Singapore, Singapore; 4 School of Civil and Environmental Engineering, Nanyang Technological University, Singapore, Singapore; 5 School of Materials Science and Engineering, Nanyang Technological University, Singapore, Singapore; 6 School of Chemical and Biomedical Engineering, Nanyang Technological University, Singapore, Singapore; 7 School of Biotechnology and Biomolecular Sciences, The University of New South Wales, Sydney, NSW, Australia; 8 Department of International Health, Immunology and Microbiology, Panum Institute, University of Copenhagen, Copenhagen, Denmark; University Paris South, France

## Abstract

The biosynthesis of the redox shuttle, phenazines, in *Pseudomonas aeruginosa*, an ubiquitous microorganism in wastewater microflora, is regulated by the 2-heptyl-3,4-dihydroxyquinoline (PQS) quorum-sensing system. However, PQS inhibits anaerobic growth of *P. aeruginosa*. We constructed a *P. aeruginosa* strain that produces higher concentrations of phenazines under anaerobic conditions by over-expressing the PqsE effector in a PQS negative Δ*pqsC* mutant. The engineered strain exhibited an improved electrical performance in microbial fuel cells (MFCs) and potentiostat-controlled electrochemical cells with an approximate five-fold increase of maximum current density relative to the parent strain. Electrochemical analysis showed that the current increase correlates with an over-synthesis of phenazines. These results therefore demonstrate that targeting microbial cell-to-cell communication by genetic engineering is a suitable technique to improve power output of bioelectrochemical systems.

## Introduction

Microbial fuel cells (MFCs) are bioelectrochemical devices in which viable electroactive biofilms (EABs) convert chemical energy directly into electrical energy [1], [2], [3]. In a typical two-chamber configuration, viable EABs in the anaerobic anode chamber oxidize organic substrates as part of its metabolic functions. The electrons liberated in the microbial breakdown process are then transferred to the extracellular electrode through direct and/or mediated charge transfer mechanisms. In this manner, the electrons can flow through an external circuit across an electrical load, thus producing electrical power. The protons diffuse across a proton exchange membrane to the aerobic cathode chamber, where a biological or chemical catalyst promotes oxygen reduction and the formation of water to maintain overall charge balance.

MFCs also enable simultaneous wastewater treatment and bioelectricity generation [4], [5]. However, the main limitation for full-scale implementation of this technology is the slow anodic electron transfer processes occurring at the biofilm-electrode interface [6]. Common electrochemically active bacteria, such as *Geobacter sulfurreducens* [7], [8] and *Shewanella oneidensis* MR-1 [9], [10], have been frequently used to produce bioelectricity in MFCs. Extracellular electron transfer occurs either through membrane associated cytochromes [11], secretion of soluble electron shuttles or mediators [12], [13], [14], or physical conductive appendages, termed as nanowires [15], [16], [17].

*P. aeruginosa* is a microorganism commonly found in wastewater treatment systems. It produces phenazines as soluble redox mediators to enhance electron transport [18], [19]. Phenazine biosynthesis is positively regulated by the *Pseudomonas* quinolone signal (PQS) system [20], [21]. However, there are several factors which limit the bioelectricity generation of *P. aeruginosa* in bioelectrochemical applications. Firstly, the PQS system remains largely inactive due to the requirement of oxygen and thus this limits phenazine biosynthesis [22]. Secondly, PQS signaling represses anaerobic growth of *P. aeruginosa* and other co-culture bacterial species [23], [24].

Recent studies showed that synthetic PqsE can regulate phenazine biosynthesis without the presence of PQS signaling [21], [25]. In this study, we constructed a PQS defective but phenazine over-producing *P. aeruginosa* strain by introducing a synthetic PqsE gene into a Δ*pqsC* mutant. We measured anodic current production in electrochemical cells, electrical power output in laboratory-scale MFCs and quantified phenazine produced by the Δ*pqsC*+sPqsE strain through high resolution accurate mass spectrometry (MS). The engineered strain showed much higher electrochemical activity and concentrations of pyocyanin in MFCs. We suggest that manipulation of the microbial signaling system might provide novel strategies to improve the power output of bioelectrochemical devices.

## Materials and Methods

### Bacteria and Growth Conditions

Wild-type *P. aeruginosa* PAO1 was used in this study [26]. *Escherichia coli* strains MT102 and DH5a were used for standard DNA manipulations. Luria–Bertani (LB) medium [27] was used to cultivate both *E. coli* strains. *P. aeruginosa* was cultivated at 37°C with modified ABTG medium (made up of 15.1 mM ammonium sulfate, 33.7 mM sodium phosphate dibasic, 22.0 mM potassium dihydrogen phosphate, 0.05 mM sodium chloride, 1 mM magnesium chloride hexahydrate, 100 µM calcium chloride anhydrous, 1 µM iron (III) chloride) [28] supplemented with 0.5% (w/v) glucose. Selective media were supplemented with ampicillin (Ap; 100 mg/litre), gentamicin (Gm; 60 mg/litre) and carbencillin (Carb; 200 mg/litre).

### Engineering of PQS Signalling Mutant

**Figure 1:**
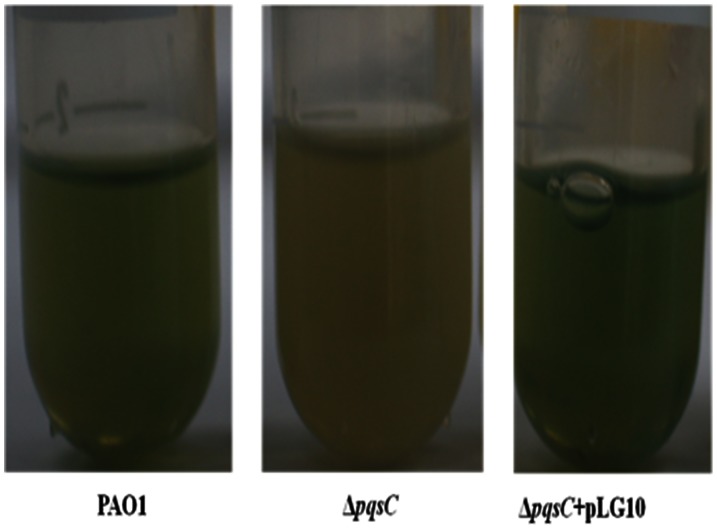
Pyocyanin production by *P. aeruginosa* PAO1, ΔpqsC and ΔpqsC+pLG10.

The *P. aeruginosa* Δ*pqsC* mutant was identified from a transposon mutagenesis library screen, which was constructed by using the Mariner transposon vector pBT20, as previously described [29]. Transconjugants carrying transposon insertion were picked from the selective plates and inoculated into microtiter tray wells containing LB medium by using a Qpix2 robot (Genetix). Mutants that produced reduced levels of pyocyanin were selected and saved for further analysis. For identification of the transposon insertion site, the sequence flanking the Mariner transposon in selected mutants was identified by arbitrary polymerase chain reaction (PCR), as previously described [30] but with the following TnM specific primers: Rnd1-TnM 5′-GTG AGC GGA TAA CAA TTT CAC ACA G, Rnd2-TNm 5′-ACA GGA AAC AGG ACT CTA GAG G. Sequencing was performed by Macrogen (Seoul, Korea) with the TnM specific primer: TnMseq 5′-CAC CCA GCT TTC TTG TAC AC. Its phenotype could be rescued by complementation with the provision of the *pqsA-E* operon *in trans* (on plasmid pLG10 [20], kindly provided by Dr. Colin Manoil) (Figure 1). A Δ*pqsC* mutant was identified in the screen and the pyocyanin deficient phenotype could be restored by provision of the *pqsA-E* operon *in trans* on plasmid pLG10. The synthetic sPqsE over-expression vector [25] was kindly provided by Dr. Paul Williams. The sPqsE vector was transformed into PAO1 and Δ*pqsC* via electroporation and selected on LB agar supplemented with 200 mg/liter carbencillin.

### MFC Set-up and Electrochemical Measurements

All materials were used as received unless otherwise stated. Carbon felt (3.18 mm thickness) and stainless steel pinch clamps (#28) were purchased from VWR Singapore Pte. Ltd. Titanium wire (0.25 mm diameter), Nafion® N117 and serrated silicone septa (18 mm O. D.) were purchased from Sigma-Aldrich, Singapore. Glass tubes (17 mm O. D. ×1.8 mm wall thickness) were purchased from Ace Glass, Inc. (Vineland, NJ) to form the anode and cathode chambers of the MFCs.

Dual chamber U-tube MFCs were constructed as reported previously [31], [32]. Two 90° O-ring-groove-to-plain-end glass tubes were separated from each other by a piece of Nafion® N117 proton exchange membrane. The joints of the glass tubes were greased and sealed against a circular piece of Nafion® membrane (diameter of 2 cm). The whole assembly was held in place and tightened with a stainless steel pinch clamp. Carbon felt electrodes were cut to 2 cm×5 cm dimensions (width×length) and woven at one end with titanium wire to which electrical leads would later be attached, and the electrodes were then seated inside the glass tubes. Prior to MFC operation, the devices were filled with ultrapure water and autoclaved to sterilize the internal components in the devices. After sterilization and decanting off the ultrapure water, the anode and cathode chambers were each filled with sterile ABTG growth medium. 1 mL of live cell culture solution (OD_600_ = 2) was inoculated into the anode chamber only. The final total volume of solution in each of the anode and cathode chambers was 20 mL. The anode chamber was sealed with a silicone septum through which the titanium wire was threaded, while the cathode chamber was loosely capped with an inverted glass scintillation vial to provide an aerobic environment. The cathode electrodes were only partly submerged in the catholyte to allow for an ‘air-wicking’ aerobic configuration. The electrodes were then connected to a 1 kΩ resistor and voltage measurements across the resistors were recorded at a rate of 1 point per minute using an eDAQ e-corder® data acquisition system (Bronjo Medi, Singapore) equipped with Chart® software. Data collection started immediately after inoculation of the devices. Two sets of MFCs were kept inside incubators set to 36°C and 30°C for up to approximately 3 days.

### Electrode Preparation in Electrochemical Cells (ECs)

Commercial carbon felt (3.18 mm thickness) was cut into 1 cm×1 cm electrodes. The carbon felt electrodes were kept overnight in 1 M HCl, then stored in deionized (DI) water, prior to assembly of ECs. This pretreatment increased wettability and favored biomass attachment on the electrodes.

### Biofilm Growth in ECs

1 mL of the *P. aeruginosa* cell suspension (OD_600_ = 1) was added to the ECs which were already filled with 9 mL of supplemented ABTG growth medium. All electrochemical experiments were performed in technical triplicates.

### Electrochemical Setup and Analyses

Conical ECs (BASi Analytical Instruments, USA) of 10 mL working volume with a configuration of three electrodes were used as previously described [33]. The Ag/AgCl reference electrodes (BASi Analytical Instruments, USA) were connected to the ECs via 100 mM KCl salt bridges ending in 3 mm Vycor glass membranes (BASi Analytical Instruments, USA). All electrochemical potentials are described versus Ag/AgCl reference electrode. A 0.1 mm titanium coil (VWR, Singapore) was used as the counter electrode. The working electrodes were attached to the potentiostat via titanium wire, nylon screws and nuts (Small Part, USA). The ECs were stirred by magnetic stirrers at a constant speed of 150 rpm. The headspaces of the ECs were continuously flushed with humidified, sterile nitrogen gas. The ECs were maintained at 30°C throughout the experiment and the working electrodes were poised at 400 mV. The ECs were connected to a VSP 5-channel potentiostat (Bio-Logic, France). Chronoamperometry (CA), cyclic voltammetry (CV), and differential pulse voltammetry (DPV) were used to analyze the *P. aeruginosa* biofilms formed at the working electrodes. CV scan rate was 10 mV/s; DPV parameters were: pulse height = 50 mV; pulse width = 200 ms; step height = 2 mV; step time = 400 ms; scan rate = 5 mV/s. CV and DPV were performed at the beginning of the experiment and after approximately 20 hours.

### Mass Spectrometry Analysis

Mass spectrometry analysis for the identification and quantification of the pyocyanin containing samples was performed in the positive mode on the Orbitrap Velos Pro (Thermo Scientific, USA) interfaced with either high performance liquid chromatography (HPLC) pump (Accela 1250, Thermo Scientific, USA) or a robotic nanoflow ion source, Nanomate (Advion Biosciences, Ithaca, NY). Each sample was diluted 1 to 10 in MilliQ water. For identification, 5 µL aliquots from the samples were injected into the mass spectrometer by the Nanomate robotic device operating in the direct infusion mode. For quantification, 20 µL aliquots were injected by the Open Accela autosampler (Thermo Scientific, USA) into an Accela 1250 series HPLC and further separated on a Thermo Scientific Hypersil Gold C18 column (2.1 mm×50 mm) by a 12 minute gradient program with a solvent flow rate of 150 µL/min. Solvent A was 1% (v/v) acetonitrile (LC-MS grade, Fluka, Switzerland) in water with 0.1% (v/v) formic acid (Fluka, Switzerland). Solvent B was 100% acetonitrile with 0.1% formic acid. A linear gradient from 0% to 10% B was applied during the first 5 minutes. The concentration of solvent B was then linearly ramped to 90% in 1 minute and maintained at 90% for 1 more minute. Concentration of solvent B was then dropped to the initial concentration of 0% during 1 minute and the column was allowed to re-equilibrate for an additional 4 minutes. Each sample was measured in 3 technical replicates. Aliquots were collected from all MFCs kept at 30°C.

Quantization was done using extracted ion chromatograms (XICs). Selected signals corresponding to ions of interest were extracted along the LC-MS run based on their exact m/z values with a deviation of 3 ppm. Extracted LC peak areas were integrated at a corresponding retention time (RT) by the XCalibur software (Thermo Scientific, USA). Concentrations of pyocyanin were calculated, based on the values of the XICs relative to the previously constructed calibration curve within its linearity range and adjusted to initial dilution.

## Results

### Construction of PQS Defective but Phenazine Over-producing *P. aeruginosa* Strain

**Figure 2:**
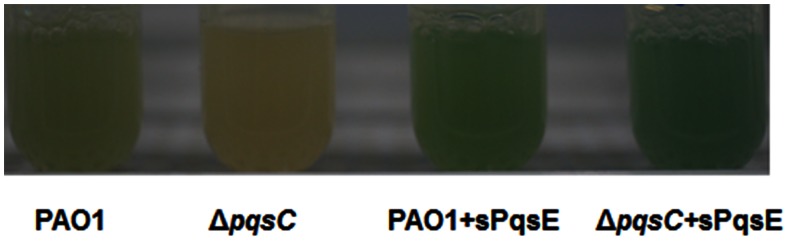
Observation of pyocyanin production in *P. aeruginosa* strains. *P. aeruginosa* strains were grown in LB medium for 18 h, and aliquots of respective cultures were recorded to document the production of the blue-green pigment, pyocyanin.

As reported previously, the PQS defective Δ*pqsC* mutant produced very low concentrations of the redox-active phenazine compound, pyocyanin (Figure 2) while both PAO1+sPqsE and Δ*pqsC*+sPqsE overproduce pyocyanin, as compared to the wild-type PAO1 strain [21], [25].

### Increase in Current Output by PQS Defective but Phenazine Over-producing Strain in MFCs

**Figure 3:**
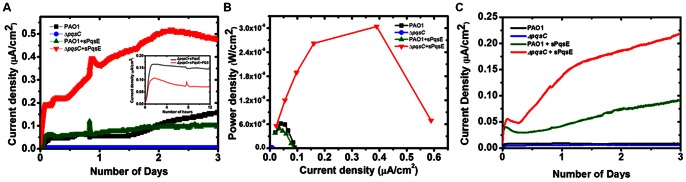
Electrical output of MFCs inoculated with various microbial strains. (A) Graph of averaged current density generated as a function of time by various microbial strains kept in incubator at 36°C. (B) Graph of MFC power curves with various microbial strains at 36°C. (C) Graph of averaged current density generated as a function of time by various microbial strains kept in incubator at 30°C.

To evaluate the hypothesis that genetic engineering can manipulate phenotypic expression leading to over-production of pyocyanin electron shuttles, dual chamber U-tube MFCs were employed as the test platform to study the electrical output of the various engineered bacterial strains upon inoculation to the anodic chamber. Figure 3A is an illustration of the temporal profiles of the averaged current density generated by various engineered microbial strains kept in an incubator at 36°C over a period of 3 days. It is evident that the maximum current density of 0.5 µA/cm^2^ achieved by the genetically engineered bacterial strain Δ*pqsC*+sPqsE after 3 days of operation was approximately five times higher than the rest of the strains. Upon bacterial strain inoculation, it was also observed that there was an immediate initial increase in current density by the Δ*pqsC*+sPqsE strain, as compared to the other strains. The averaged power curves measured on Day 2 were presented in Figure 3B. Peak power density (red trace) was highest for MFCs inoculated with Δ*pqsC*+sPqsE strain. These observations support the hypothesis that enhancing production of electron shuttles in bacterial strains by genetically modifying the quorum sensing system can improve current production. The averaged power curve of PAO1+sPqsE, however, was similar to that of PAO1 strain. It should be noted that both averaged power curves were significant lower than the averaged power curve of Δ*pqsC*+sPqsE strain. The lower level of power generation from the PAO1+sPqsE strain compared to the Δ*pqsC*+sPqsE strain might be due to the fact that the PAO1+sPqsE strain was still able to synthesize small amount of PQS in the MFCs, which was not fully anaerobic initially. The PQS could inhibit anaerobic growth of *P. aeruginosa* [23], [24]. To test this hypothesis, we added purified PQS (25 µM, synthesized as described before [34] by Prof. Paul Williams’s group) into the MFC containing Δ*pqsC*+sPqsE bacterial strain. Addition of PQS significantly reduced power generation by the Δ*pqsC*+sPqsE strain in MFCs (inset of Figure 3A). Another set of MFCs were also inoculated with the same bacterial strains and monitored at 30°C for up to approximately 3 days. The graph of the averaged current density versus time generated by various engineered microbial strains is shown in Figure 3C. It is evident that the trend in power output for genetically engineered microbial strains still hold true. In terms of power output in MFCs, the power output performance is: Δ*pqsC*+sPqsE >PAO1+sPqsE >PAO1> Δ*pqsC*.

In order to further validate our hypothesis that the microbial produced redox mediator, pyocyanin, is the electron shuttle, which increases charge transfer within the biofilm, liquid-chromatography-mass spectrometry technique was employed to characterize and quantify the presence and concentration of pyocyanin respectively. Aliquots from the anode chamber of the MFCs were collected and filtered through a 0.22 µm syringe filter to obtain the solution for analysis. From the investigation, the concentration of pyocyanin produced by the genetically engineered bacterial strain of Δ*pqsC*+sPqsE was 165 nM (with standard deviation of 50 nM), whereas the pyocyanin concentration from wild type PAO1 was 72 nM (with standard deviation of 45 nM). The Δ*pqsC* strain produced 58 nM of pyocyanin (with standard deviation of 36 nM). This data correlates and corresponds well with other observations from power output of MFCs and electrochemical analysis. Pyocyanin concentration and the current produced were highest in Δ*pqsC*+sPqsE and the least in the Δ*pqsC* strain.

### Electrochemical Analysis of *P. aeruginosa* Early Stage Biofilms

**Figure 4:**
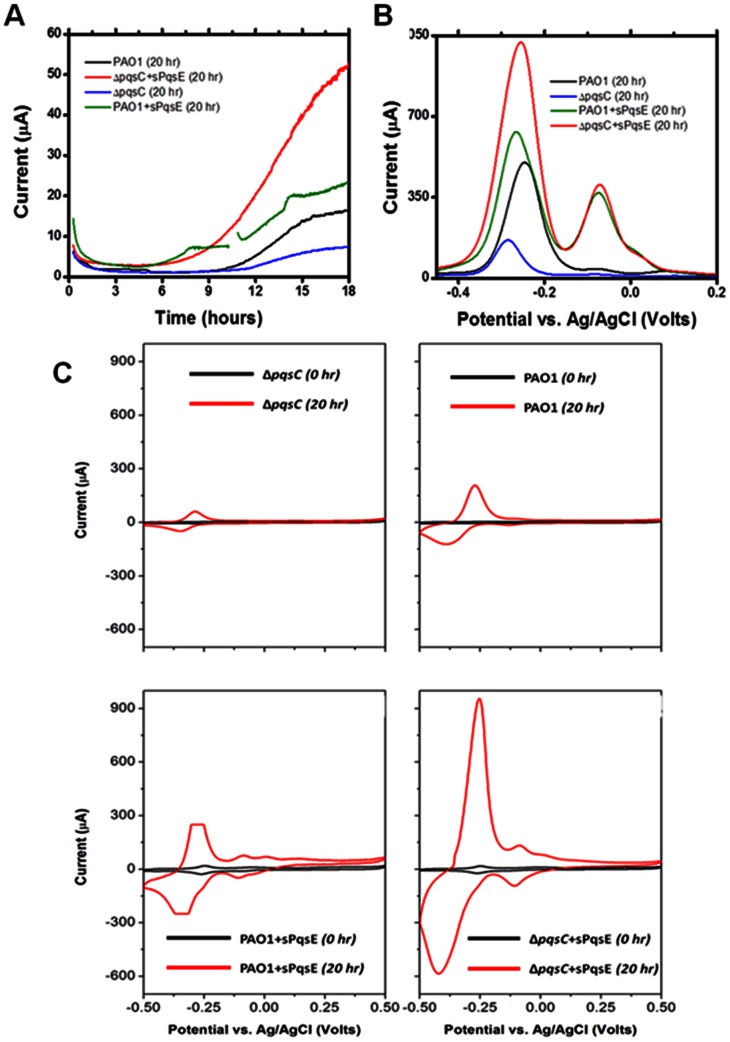
Electrochemical analyses of various microbial strains. (A) Chronoamperometry of *P. aeruginosa* biofilms poised at potential of 400 mV vs. Ag/AgCl. (B) Differential pulse voltammetry of *P. aeruginosa* biofilms after 20 hr of growth poised at potential of 400 mV vs. Ag/AgCl. (C) Representative CVs of the four *P. aeruginosa* strains used in this study. The CV of the PAO1+sPqsE strain at 20 hr was truncated due to an off-scale condition of the potentiostat.

We tested the genetically engineered *P. aeruginosa* strains in potentiostat-controlled electrochemical cells (ECs). The working electrode was poised at 400 mV vs. Ag/AgCl to serve as a non-limiting electron acceptor. The growth medium did not contain any soluble electron acceptor. As previously reported, anaerobic conditions could completely inhibit PQS generation even from PAO1 wild-type [35]. We expected this cultivation model to improve power generation of the PAO1+sPqsE strain. The microbial produced phenazines increased the electron transfer from the cytoplasm to the electrode through the biofilm. After an initial lag phase, which was likely due to the expression of the enzymatic machinery required for anaerobic growth, chronoamperometry showed that the rates of extracellular electron transport (EET) for all the tested strains increased with time, as measured by the increase of anodic (oxidation) currents (Figure 4A). The slope of current vs. time decreased in the order: Δ*pqsC*+sPqsE >PAO1+sPqsE >PAO1> Δ*pqsC*. Current increased until a plateau was reached after approximately 20 hours. At this time, we observed an increase in the concentration of planktonic cells and the appearance of a thin biofilm at the working electrode (data not shown). As we are investigating the role of pyocyanin as an EET agent in early biofilm formation, we did not carry out long-term experiments. It should be noted that the same experiments on better-performing electrochemically active strains would have required 3–4 days to complete because of their low growth (data not shown). The rapid growth of *P. aeruginosa* under electroactive conditions allowed us to reduce the experimental time and the likelihood of contamination. DPV results in Figure 4B provided greater insight into the concentration of redox active species at the electrode surface. The current production measured in the chronoamperometry analysis correlates well with the height of the DPV peak at −270±10 mV vs. Ag/AgCl, indicating that the corresponding redox species contribute mostly to the overall EET process at the microbe-electrode interface. An additional peak at higher potential was observed for the two PqsE over-expressing mutants (Δ*pqsC*+sPqsE and PAO1+sPqsE). However, this peak does not correlate well with the current density in CA. We suggest that the main peak corresponds to pyocyanin, based on a previous report [36]. The second peak at a higher potential may be attributed to a pyocyanin derivative or another phenazine compound expressed by the mutants. CV analysis at 10 mV/s in Figure 4C showed that the two major electroactive species detected in the DPV analysis undergo a partially irreversible reduction, as the cathodic peak was much smaller and broader than the anodic one. CV analyses were performed at 10 mV/s immediately after inoculation (0 hours) and after 20 hours of growth. Each scan was repeated 3 times. The second and third cycles were very similar, hence only the third cycle is reported. All cycles were obtained under non-limiting electron donor (glucose) concentration. Representative CVs (n = 3) of each *P. aeruginosa* strain described in this study are shown in Figure 4C. At 0 hours, the concentration of redox active species was very low. Traces of CVs had several small peaks, which could be due to the accumulation of metabolites during overnight growth and to the medium itself (i.e., amino acids). As the concentrations of planktonic and attached cells increased, a major redox peak with a midpoint potential of −324±13 mV vs. Ag/AgCl appeared. In addition, a second smaller peak appeared at −90±1 mV vs. Ag/AgCl, but was observed only in the PAO1+sPqsE and Δ*pqsC*+sPqsE mutants. The I_p, a_/I_p, c_ value for the major peak was less than 1, indicating that the corresponding redox species has undergone a partially irreversible electron transfer reaction. The CV traces did not show any evidence of turnover electron transfer. This suggests that the redox reactions are controlled by the diffusion of the microbial produced pyocyanin in the biofilm formed at the electrode.

## Discussion

Current research aims to increase extracellular electron transfer rates at the electrodes by a variety of methods, such as a) electrode modification to encourage biofilm growth and improve charge transfer [37], [38], [39]; b) optimization of MFC bioreactors to minimize limiting conditions [13], [40]; c) manipulation of EAB ecology, microstructure and chemistry [41], [42]; d) genetic engineering to overproduce microbial redox shuttles [36].

Quorum sensing has recently been shown to improve MFC performance through regulation of phenazines production in these bioelectronic devices [43], hence it is an ideal methodology to exploit, so as to improve MFC performance as quorum sensing can be manipulated either genetically or chemically. In a recent study, Yong et al. showed that genetic enhancement of the *P. aeruginosa rhl* quorum sensing circuit can improve MFC performance [36]. However, many of the *P. aeruginosa* quorum sensing regulated products are virulence factors, which can inhibit the growth of co-cultivated microorganisms and even *P. aeruginosa* itself [44]. For example, 2-heptyl-4-hydroxyquinoline (HHQ) from *P. aeruginosa* PQS signaling pathway has antibacterial activity against *Vibrio anguillarum*, *Staphylococcus*, *Candida albicans* and *Vibrio harveyi* [45], [46]. Another quorum sensing regulated product, rhamnolipid, also shows broad-spectrum antimicrobial activity against Gram-positive and Gram-negative bacteria [47]. Quorum sensing was also shown to regulate synthesis of biofilm extracellular polymeric substance (EPS) [48], [49], which was demonstrated to reduce the conductivity of biofilms in MFCs [50].

In this study, we modified the PQS quorum sensing signaling circuit by abolishing PQS signaling and over-expressing a synthetic sPqsE. The abolishing of PQS signaling can remove the negative impact of PQS quorum sensing on anaerobic growth, while over-expression of PqsE could maintain the high level of phenazine production. This strategy significantly improves the performance of *P. aeruginosa*-based MFCs and the current production in potentiostat-controlled electrochemical cells. The over-production of pyocyanin increases EET and may be of interest for mixed culture bioelectrochemical devices. As phenazine compounds from *P. aeruginosa* are able to enhance MFC performance by some bacterial species [18] while the PQS is known to inhibit the growth of other bacterial species [23], [24], further studies will be carried out to study the synergy between our engineered *P. aeruginosa* strains with other microbial species to further evaluate the impact of PQS on power generation by functional microbial communities.

### Conclusions

We employed a rationally designed genetic engineering strategy to improve EET processes in *P. aeruginosa* biofilms via over-expression of the phenazine redox mediators. Current density output in laboratory-scale MFCs and ECs increased five times with the bacterial strain which over-produces pyocyanin. The pyocyanin overproduction was verified by electrochemical analyses and quantitative high resolution accurate mass spectrometry. Our results show that genetic engineering strategies and the synthetic biology approach enable over-expression of electron shuttles and enhancement of EET rates at the microbe-electrode interface. Our findings may lead to the design of high-performance multispecies MFCs for energy recovery from wastewater treatment.
